# Social phobia and other syndroms in late-onset psychosis

**DOI:** 10.1192/j.eurpsy.2022.465

**Published:** 2022-09-01

**Authors:** V. Pochueva, M. Savina, V. Sheshenin

**Affiliations:** FSBSI “Mental Health Research Centre”, Geriatric Psychiatry, Moscow, Russian Federation

**Keywords:** old age, social phobia, late onset schizophrenia, complicated grief

## Abstract

**Introduction:**

Anxiety disorders are frequent comorbidity in patients with schizophrenia. Their frequency in late-onset psychosis is unknown.

**Objectives:**

We aimed to study life-time anxiety disorders as well as other psychiatric pathology in patients with late-onset schizophrenia (LoS) and late-onset delusional disorder (LoDD).

**Methods:**

42 patients with LoS (age 64,1±9,2, 12% males) and 15 patients with LoDD (n=15; age 69,9±10,9, 14% males ) underwent clinical assessment (PANSS, HDRS-17), cognitive examination (MMSE, MoCA), structured interviewing on life-time pathology. Control group included 24 subjects with no signs of depression or psychosis (age 58,1±10,8, 50% males). Kruscal-Wallis and χ^2^statistic was used.

**Results:**

Patients with LoS and LoDD had higher frequency of social phobia (50% and 30% compared to 16,7 in control group, χ^2^ (2)= 6,834, p= 0,033) and more animal phobias (KW χ^2^ (2)=15,536, p<0,001). The number of habitual anxiety reaction was increased in LoS but not LoDD patients compared to controls (KW χ^2^ (2)=7,275, p= 0,026) with more severe motor (KW χ^2^ (2)=8,516, p= 0,014), vegetative (KW χ^2^ (2)=8,633, p= 0,013 and ideatory symptoms (KW χ^2^ (2)=6,969, p= 0,031) of anxiety. There was no group difference in life-time frequency of depression. Patients and controls had similar rate of complicated grief, but controls had often no reaction in response to loss and LoS patients responded to loss with manifestation of psychosis (χ^2^ (6)= 14,473, p= 0,025).

**Conclusions:**

Social phobia and other anxiety disorders are frequent in late-onset psychosis. Symptoms of generalized anxiety are more specific to LoS than to LoDD.

**Disclosure:**

No significant relationships.

